# Long-term potentiation in hippocampal oriens interneurons: postsynaptic induction, presynaptic expression and evaluation of candidate retrograde factors

**DOI:** 10.1098/rstb.2013.0133

**Published:** 2014-01-05

**Authors:** Elizabeth Nicholson, Dimitri M. Kullmann

**Affiliations:** UCL Institute of Neurology, University College London, Queen Square, London WC1N 3BG, UK

**Keywords:** long-term potentiation, interneurons, anti-Hebbian

## Abstract

Several types of hippocampal interneurons exhibit a form of long-term potentiation (LTP) that depends on Ca^2+^-permeable AMPA receptors and group I metabotropic glutamate receptors. Several sources of evidence point to a presynaptic locus of LTP maintenance. The retrograde factor that triggers the expression of LTP remains unidentified. Here, we show that trains of action potentials in putative oriens-lacunosum-moleculare interneurons of the mouse CA1 region can induce long-lasting potentiation of stimulus-evoked excitatory postsynaptic currents that mimics LTP elicited by high-frequency afferent stimulation. We further report that blockers of nitric oxide production or TRPV1 receptors failed to prevent LTP induction. The present results add to the evidence that retrograde signalling underlies *N*-methyl-d-aspartate (NMDA) receptor-independent LTP in oriens interneurons, mediated by an unidentified factor.

## 1. Introduction

Plasticity of glutamatergic synapses onto hippocampal interneurons has been proposed to play several roles, including stabilizing network excitability and preserving the fidelity of spatio-temporal processing in the face of long-term potentiation (LTP) at synapses among excitatory neurons [1–3]. LTP and long-term depression (LTD) in the inhibitory circuitry may also affect the input–output relationship of principal cells in qualitatively different ways than that achieved by plasticity restricted to excitatory neurons. Plasticity of inhibition may have further adaptive roles particular to specific types of interneurons [4]. For example, LTP of glutamatergic synapses on somatostatin-positive interneurons located in stratum oriens, which tend to innervate targets in strata radiatum and lacunosum-moleculare, is likely to enhance CA1 inputs from CA3 by disinhibiting Schaffer-collateral-associated interneurons, while concomitantly inhibiting extrahippocampal perforant path inputs to the apical dendrites of CA1 pyramidal neurons [5].

Induction of LTP at glutamatergic synapses on several types of interneurons in stratum oriens is independent of *N*-methyl-d-aspartate (NMDA) receptors, but requires Ca^2+^ influx through Ca^2+^-permeable α-amino-3-hydroxy-5-methyl-4-isoxazolepropionic acid (AMPA) receptors as well as activation of group I metabotropic glutamate receptors (mGluRs) [6–12]. Among these are interneurons with dendrites running parallel to stratum pyramidale that project an axon to stratum lacunosum-moleculare [13]. Oriens-lacunosum-moleculare (O-LM) cells can be further recognized by their regular firing pattern upon injection of depolarizing current, voltage sag upon injection of hyperpolarizing current and expression of somatostatin and mGluR1a [14–17]. An efficient LTP induction stimulus is to hyperpolarize the postsynaptic membrane potential while stimulus trains are delivered to axon collaterals of pyramidal neurons running in the alveus. This ‘anti-Hebbian’ LTP protocol has been hypothesized to maximize Ca^2+^ influx via rectifying AMPA receptors [8,11], although nicotinic receptors have also been proposed to play a role [18,19].

NMDA receptor-independent LTP in stratum oriens interneurons is associated with a decrease in the rate of failures of stimulus-evoked transmission, a decrease in the paired-pulse ratio (PPR) of excitatory postsynaptic currents or potentials (EPSC/Ps), a decrease in normalized coefficient of variation (equivalently, an increase in the parameter CV^−2^) and an increase in estimated AMPA receptor occupancy [8,10,13]. All these observations strongly argue for a presynaptic locus of expression of LTP, in striking contrast with conventional NMDA receptor-dependent LTP at glutamatergic synapses on pyramidal neurons [20]. A recent report has however suggested that anti-Hebbian LTP in parvalbumin-positive interneurons may also be expressed postsynaptically [21].

The postsynaptic induction requirements and evidence for presynaptic expression of LTP on O-LM cells and other interneurons in stratum oriens imply the existence of a retrograde messenger. Identification of this factor has been hampered by the relative difficulty of eliciting LTP while recording in the whole-cell configuration of the patch-clamp method [8,11]. We therefore first set out to identify recording conditions where LTP could be reliably elicited in whole-cell mode and systematically interleaved control experiments throughout this study whenever pharmacological interventions were applied. We confirm that LTP requires postsynaptic Ca^2+^ and show that it can be induced by trains of action potentials elicited in the interneuron alone, implying that multiple sources of Ca^2+^ may converge on the induction trigger. We provide further evidence of presynaptic expression, and test two candidate retrograde factors.

## 2. Material and methods

### (a) Brain slicing

Horizontal brain slices were prepared from postnatal day 21–25 mice in accordance with the UK Animals (Scientific Procedures) Act 1986. Slices (300 μm thick) were cut using a vibrating microtome (Leica VT 1200) in an ice-cold solution bubbled with 95% O_2_ and 5% CO_2_, containing (in mM): sucrose, 75; NaCl, 80; KCl, 2.5; CaCl_2_, 0.5; MgCl_2_, 7; NH_2_PO_4_, 1; NaHCO_3_, 25 and glucose, 10. Slices were warmed to 32°C for 15 min, and then stored at room temperature in a solution containing (in mM): NaCl, 119; KCl, 2.5; CaCl_2_, 0.5; MgSO_4_, 1.3; NaH_2_PO_4_, 1.25; NaHCO_3_, 25 and glucose, 10, bubbled with 95% O_2_ and 5% CO_2_.

### (b) Electrophysiology

Slices were anchored in a recording chamber mounted on the stage of an upright microscope (BX51WI, Olympus) and visualized with a ×20 water immersion objective with infrared differential interference contrast optics. Slices were continuously perfused at a rate of 3 ml min^−1^ with a carbogen-bubbled solution containing (in mM): NaCl, 119; KCl, 2.5; CaCl_2_, 2.5; MgSO_4_, 1.3; MgCl_2_, 2; NaH_2_PO_4_, 1.25; NaHCO_3_, 25; glucose, 10. To block NMDA, gamma-aminobutyric acid A (GABA_A_) and GABA_B_ receptors, we routinely added 50 μM d-aminophosphonovalerate (AP5), 100 μM picrotoxin and 1 μM CGP 52432. The pipette solution contained (in mM): K-gluconate, 80; NaCl, 8; KOH-HEPES, 20; EGTA, 0.2 or BAPTA, 25; biocytin, 0.5. Polyamines were deliberately omitted to relieve the voltage dependence of AMPA receptor conductance.

Interneurons within stratum oriens of CA1 with dendrites running parallel to stratum pyramidale were patch-clamped with 4–5 M*Ω* glass pipettes, to achieve an access resistance of less than 20 M*Ω*. Data were acquired using a Multiclamp 700 B amplifier (Molecular Devices), low-pass filtered (4–5 kHz) and digitized at 10–20 kHz (National Instruments).

**Figure 1.**
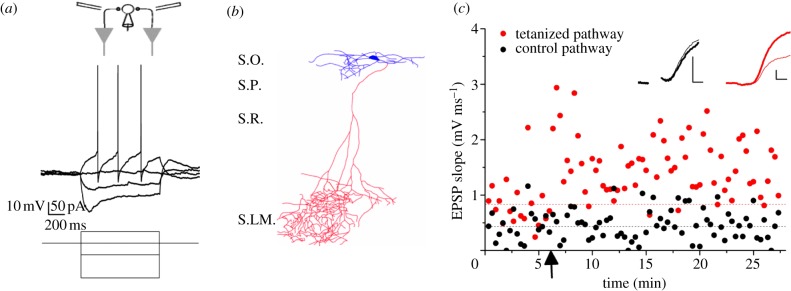
LTP in an O-LM cell. (*a*) Electrical properties of an identified O-LM cell. Top: schematic showing recording pipette and electrical stimulation designed to activate pyramidal neuron axon collaterals innervating the interneuron. Below: response to hyper- and depolarizing currents, showing a pronounced voltage sag upon −150 pA current injection and regular firing with deep afterhyperpolarization (AHP) upon +50 pA current injection. (*b*) Reconstructed interneuron filled with biocytin, showing horizontally orientated dendrites (top) contained within stratum oriens (S.O.) and an axon passing through strata pyramidale and radiatum, (S.P. and S.R.) arborizing in stratum lacunosum-moleculare (S.L.M.). (*c*) LTP experiment in the same cell shown in (*a*,*b*). Tetanic stimulation (2 × 100 Hz, 1 s) was delivered at the time indicated by the arrow to one pathway (red, grey in printed version). The tetanized pathway EPSP slope increased approximately 100% while the control pathway was unaffected. Insets: averaged EPSPs in the two pathways before (thin lines) and 20 min after (thick lines) tetanic stimulation. (The stimulus artefact was blanked for the control pathway.) Scale bars, 1 mV, 2 ms. (Online version in colour.)

Neurons were held in current clamp mode, and current was injected when needed to maintain the membrane potential between −70 and −75 mV. Passive membrane properties and firing patterns were tested with hyperpolarizing and depolarizing steps. Cells that did not display a voltage sag, deep afterhyperpolarization (AHP) and regular firing pattern typical of O-LM cells (figure 1*a*) were discarded.

Stimuli were delivered via two concentric bipolar electrodes, connected to constant current isolated stimulators (Digitimer), positioned in the alveus/stratum oriens border, 100–300 μm either side of the recorded interneuron. The stimulus duration was 100 μs and its intensity was adjusted between 20 and 320 μA to elicit an EPSP with peak amplitude of approximately 5 mV. Paired stimuli with an interstimulus interval of 50 ms were alternately delivered via each electrode every 20 s. The LTP induction protocol consisted of 100 stimuli at 100 Hz, delivered twice with a 20 s interval. We tested a second protocol consisting of 20 depolarizing current injections (500 pA, 500 ms, interval 5 s) to elicit trains of action potentials at a frequency of approximately 60 Hz.

LTP was studied by measuring the initial EPSP slope (2–4 ms from onset) to restrict attention to monosynaptic transmission [22–24]. For pooled data, the initial slopes of EPSPs evoked in the test and control pathways were normalized to their baselines and paired *t*-tests applied, with significance set at *p* < 0.05. Paired-pulse ratios (PPRs) were calculated in cells which exhibited LTP by averaging approximately 20 consecutive traces and expressed as the ratio of the second to the first EPSP slope. Cells with an action potential on the second pulse were omitted from PPR analysis. Failures of evoked transmission were identified by sight, and when averaged together showed no systematic deviation from the baseline, although were sometimes followed by polysynaptic EPSPs. Spontaneous EPSP frequency and amplitude were analysed using Clampfit v. 10.2 (Axon Instruments) using the first 100 events prior to LTP induction, starting at the beginning of the experiment, and 100 events 20 min after the LTP induction protocol. In both cases, the spontaneous EPSPs were obtained from periods immediately prior to stimulation. Stimulus control, and data acquisition and analysis, were achieved with custom software (National Instruments, LabView) and PClamp v. 10 software. Data are presented as mean ± s.e.m.

### (c) Anatomical analysis

Some interneurons were filled with biocytin during whole-cell recordings. Brain slices were then fixed in 4% paraformaldehyde overnight. After washing in phosphate-buffered saline (PBS), slices were incubated in PBS containing 0.3% triton and 0.1% streptavidin-alexa-488 for 3 h at room temperature, washed in PBS and mounted on coverslips with Vectashield mounting medium. Cells were visualized with an AxioImager microscope (Zeiss) and drawn in Photoshop.

### (d) Drugs

CGP 52432 and 5′-Iodoresiniferatoxin were purchased from Tocris (Bristol) and AP5 was purchased from Ascent. Picrotoxin, BAPTA and L-NNA were purchased from Sigma-Aldrich.

## 3. Results

### (a) Long-term potentiation at glutamatergic synapses onto putative oriens-lacunosum-moleculare interneurons

We recorded from cells with horizontal dendrites in stratum oriens in whole-cell current clamp mode and only continued the experiment if they showed a voltage sag with hyperpolarizing current injection, and a regular firing pattern with a deep AHP upon depolarizing current injection, typical of O-LM cells [8,13] (figure 1*a*). Of the 12 cells that were filled with biocytin and subsequently reconstructed, nine showed the typical O-LM morphology with an axon projecting into stratum lacunosum-moleculare (figure 1*b*). No axon was observed in the remaining three reconstructed cells, suggesting that it may have been cut during the slicing procedure.

We evoked monosynaptic EPSPs via two stimulation electrodes positioned at either side of the interneuron and recorded a baseline period lasting at least 5 min. Two high-frequency stimulation trains were then delivered via one electrode (100 Hz, 1 s, ×2 separated by 20 s), while stimulation via the other electrode was interrupted. The membrane potential was allowed to vary during the tetanus and the cells fired at an average rate of 34 ± 22 Hz (mean ± s.d.). In the example cell (figure 1*c*), the control pathway EPSP slope remained around 0.43 mV ms^−1^, while in the tetanized pathway it approximately doubled from 0.83 to 1.6 mV ms^−1^ (figure 1*c*).

**Figure 2.**
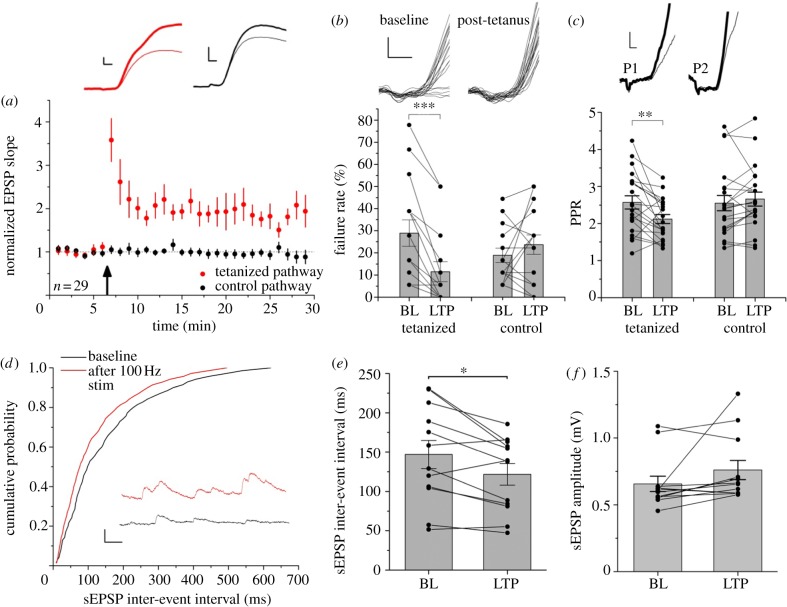
Evidence for presynaptic expression of LTP. (*a*) Summary plot showing LTP in the tetanized pathway (red, grey in printed version). EPSP slopes were normalized to baseline and remained significantly increased in the tetanized pathway for at least 20 min (*p* < 0.001, *n* = 29; paired *t*-test across pathways). Traces from an example interneuron are shown as in figure 1*c* (red, grey in printed version, tetanized; black, control; thin and thick lines, before and after tetanization of the test pathway, respectively). Scale bars, 1 mV, 2 ms. (*b*) LTP was associated with a decrease in failure rate confined to the tetanized pathway (BL, LTP, baseline and after tetanization of test pathway, respectively). Insets: sample traces in tetanized pathway from one experiment. Scale bars, 1 mV, 2 ms. (*c*) PPR was also decreased. Insets: sample average traces from an example cell (thin lines, baseline; thick lines, post-tetanus; P1, P2, first and second EPSP, respectively). Scale bars, 1 mV, 2 ms. (*d*) Spontaneous EPSPs (sEPSPs) showed a decrease in inter-event interval after LTP induction: cumulative distribution of intervals in one cell before and after LTP induction. Scale bars, 1 mV, 50 ms. (*e*) This effect was observed in data pooled from 12 cells. (*f*) The amplitudes of spontaneous EPSPs did not change significantly (*p* > 0.05). Data are shown as means ± s.e.m.; *p ≤ 0.05, **p ≤ 0.01, ***p ≤ 0.005, two-tailed paired Student's *t*-tests. (Online version in colour.)

When repeated in 29 similar experiments, we observed a robust potentiation of approximately 77% in the tetanized pathway (*p* < 0.001, paired *t*-test; figure 2*a*).

### (b) Evidence of presynaptic long-term potentiation expression

In previously unidentified stratum oriens interneurons, LTP has been proposed to have a presynaptic locus of expression [7,8]. LTP in identified O-LM cells is associated with an increase in CV^−2^ [13], consistent with an increase in glutamate release probability, although this could also be explained by postsynaptic unsilencing of AMPA receptor clusters [25]. We assessed several parameters to determine whether a presynaptic locus of LTP expression holds true for the cells recorded in this study. The failure rate decreased by approximately half in the tetanized pathway (*p* < 0.005), with no change in the control pathway (*p* = 0.29; figure 2*b*). LTP was also associated with a decrease in paired-pulse facilitation in the tetanized pathway (*p* = 0.002), with no change in the control pathway (*p* = 0.15; figure 2*c*). We also observed an increase in the frequency of spontaneous EPSPs after LTP induction, with a significant decrease in inter-event interval (*p* < 0.05; figure 2*d*,*e*) and a non-significant trend towards an increase in amplitude (*p* = 0.11; figure 2*f*). These findings argue strongly for an increase in presynaptic transmitter release.

### (c) Long-term potentiation requires postsynaptic Ca^2+^

**Figure 3.**
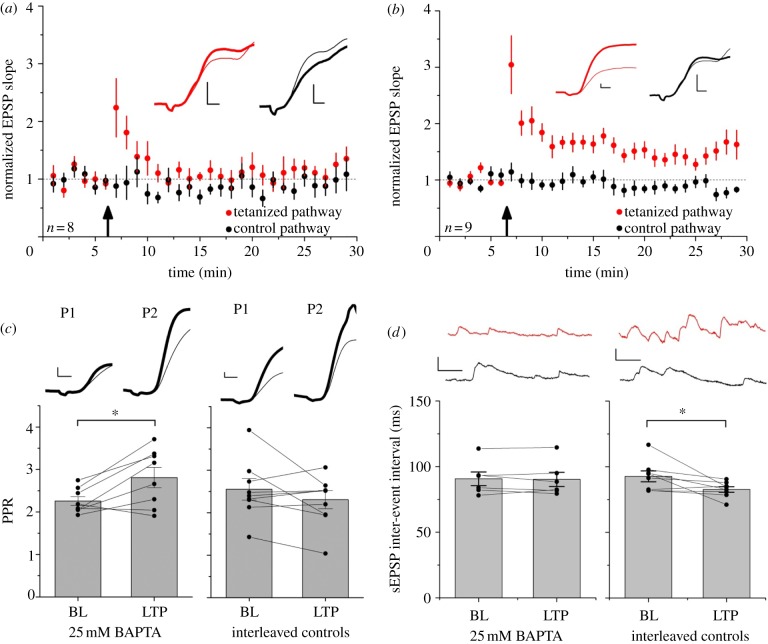
Postsynaptic Ca^2+^ chelation with 25 mM BAPTA in the pipette solution prevented LTP induction. (*a*) Average data showing the absence of LTP in cells recorded with BAPTA (*p* > 0.05), with sample average traces from one cell. (*b*) Interleaved controls showing LTP when EGTA (0.2 mM) was included in the pipette solution instead of BAPTA (*p* < 0.05). Scale bars, 1 mV, 2 ms. (*c*) PPR in tetanized pathway from cells with BAPTA included in the pipette solution displayed a significant increase 20 min after tetanus was elicited. There was a non-significant trend for the PPR of the interleaved control cells to decrease 20 min after the tetanus (*p* = 0.29). Averaged traces from example cells are shown above the pooled data (thin, baseline; thick, post-tetanus). Scale bars, 1 mV, 2 ms. (*d*) Spontaneous EPSP frequency did not increase in the cells patched with 25 mM BAPTA in the pipette solution, but did in the interleaved control cells. Raw traces from two neurons are shown above the pooled data (black, baseline; red, grey in printed version, post-tetanus). Scale bars, 1 mV, 50 ms. Data are shown as means ± s.e.m.; *p ≤ 0.05, **p ≤ 0.01, ***p ≤ 0.005, two-tailed paired Student's *t*-tests. (Online version in colour.)

LTP in horizontal oriens interneurons requires both Ca^2+^ influx through Ca^2+^-permeable AMPA receptors and activation of group I mGluRs, with evidence for both Ca^2+^ release from intracellular stores and Ca^2+^ influx through transient receptor potential channels [6,8,10–13]. We took advantage of the whole-cell recording mode to chelate intracellular Ca^2+^, by substituting BAPTA (25 mM) for EGTA (0.2 mM). Ca^2+^ chelation prevented LTP induction (*n* = 8, figure 3*a*), while interleaved control experiments continued to exhibit robust LTP (*p* = 0.005, *n* = 9; figure 3*b*). High-frequency stimulation was followed by an increase in PPR when Ca^2+^ was chelated in postsynaptic neurons (*p* = 0.02, *n* = 8; figure 3*c*). This is opposite to the pattern seen in control experiments, although the decrease in PPR in this set of experiments did not reach significance (*p* = 0.29, *n* = 9; cf. figure 2*c*). The increase in PPR was unexpected and may reflect additional presynaptic plasticity unrelated to NMDA receptor-independent LTP. Inclusion of 25 mM BAPTA also prevented the increase in spontaneous EPSP frequency (*p* = 0.76, *n* = 6; figure 3*d*), which was seen in interleaved controls (*p* = 0.05, *n* = 8; figure 3*d*).

### (d) Long-term potentiation can be induced by postsynaptic action potentials alone

**Figure 4.**
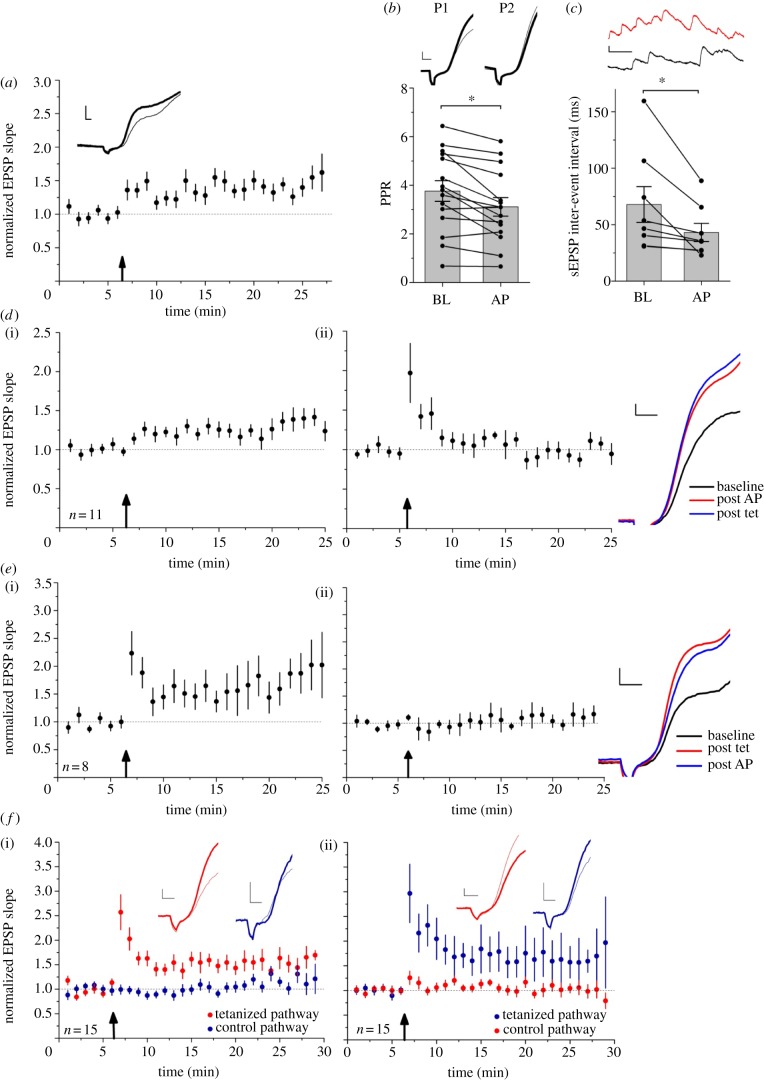
A persistent potentiation can be induced with postsynaptic action potential (AP) trains alone. (*a*) Averaged data from 15 cells showing a persistent increase in EPSP slope following trains of action potentials induced by depolarizing current injection (arrow). Sample traces from one interneuron. (*b*) Potentiation was associated with a decrease in paired-pulse ratio (scale bars, 1 mV, 2 ms) and (*c*) a decrease in sEPSP inter-event interval (scale bars, 1 mV, 50 ms). (*d*) Persistent potentiation induced by postsynaptic action potentials (i) occluded subsequent tetanus-induced LTP ((ii) EPSP slope renormalized prior to tetanic stimulation). Scale bars, 1 mV, 2 ms. (*e*) Tetanus-induced LTP (i) occluded action potential-induced potentiation ((ii) EPSP slope renormalized prior to action potential trains). Scale bars, 1 mV, 2 ms. (*f*) Effect of consecutive tetanic stimulation of two pathways with an interval of 30 min. The two pathways were renormalized to 1 prior to the second tetanization (ii). Sample traces from one experiment (stimulus artefacts blanked). Scale bars, 1 mV, 2 ms. Arrows indicate times of action potential trains or tetanic stimulation. Data are shown as mean ± s.e.m.; *p ≤ 0.05. (Online version in colour.)

We asked whether postsynaptic Ca^2+^ influx via voltage-gated channels could induce LTP. EPSPs were evoked as before, but rather than delivering tetanic stimulation, twenty depolarizing currents (500 pA for 100 ms) were injected via the recording pipette to elicit action potential trains at approximately 60 Hz. This protocol was followed by a persistent increase in EPSP slope to approximately 150% of control, which lasted for at least 20 min (*p* < 0.001, *n* = 15; figure 4*a*). The potentiation elicited by action potential trains was associated with a decrease in paired-pulse ratio (*p* = 0.004, *n* = 15; figure 4*b*) and spontaneous EPSP inter-event interval (*p* = 0.015, *n* = 8; figure 4*c*).

We asked whether the potentiation evoked by action potential trains occluded LTP. After eliciting action potential trains, tetanic stimulation failed to induce a further increase in EPSP slope (*n* = 11; figure 4*d*). Similarly, action potential trains failed to increase EPSP slope after tetanus-induced LTP (*n* = 8; figure 4*e*).

The two-way occlusion and similarity of effects on PPR and spontaneous EPSP frequency suggest that action potential trains and tetanic LTP converge on a common synaptic plasticity induction mechanism. However, an alternative possibility is that ‘washout’ of cytoplasmic constituents impaired induction of additional plasticity after a delay. To address this hypothesis, we performed a further set of experiments where high-frequency stimulation was delivered to the control pathway 30 min after inducing LTP in the test pathway. The tetanus-induced increases in EPSP slope in each pathway were not significantly different from one another (*p* = 0.92; unpaired *t*-test, *n* = 15; figure 4*f*), arguing against washout as an alternative explanation for the data summarized in figure 4*d*,*e*.

### (e) No evidence of involvement of nitric oxide and transient receptor potential vanilloid 1 channel receptors in long-term potentiation

The dependence of LTP induction on postsynaptic Ca^2+^, together with the evidence for a presynaptic locus of expression, suggests that a retrograde message signals from the postsynaptic interneuron to presynaptic glutamatergic boutons to increase glutamate release. A prominent candidate retrograde messenger that has been invoked in other examples of presynaptic LTP is nitric oxide (NO) [26–31]. Consistent with NO triggering LTP is the finding that NMDA receptor-independent LTP can be elicited in NO synthase (NOS)-positive ivy cells [12]. Direct evidence of a role of NO in LTP in unidentified stratum oriens interneurons has, moreover, been reported [32].

**Figure 5.**
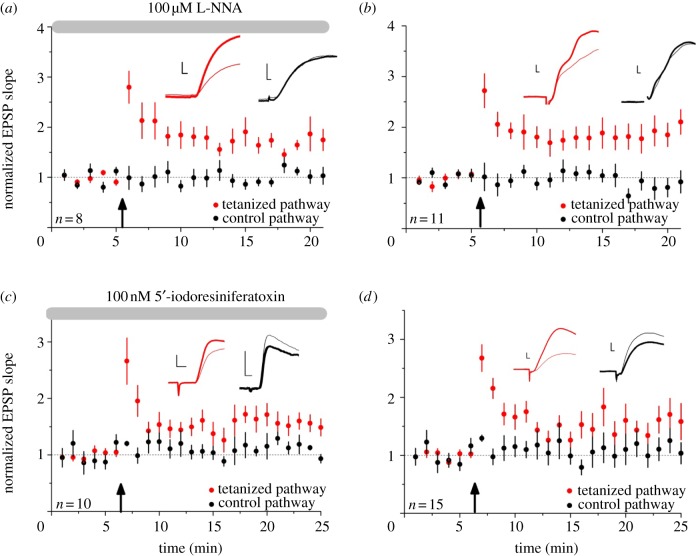
Blockade of NO or TRPV1 signalling did not prevent LTP induction. (*a*) The NOS inhibitor L-NNA failed to block tetanus-induced LTP (*p* > 0.05). (*b*) Interleaved controls (*p* > 0.05 for comparison between L-NNA and control experiments, unpaired *t*-test). (*c*) The TRPV1 antagonist 5′-iodoresiniferatoxin similarly failed to block tetanus-induced LTP. (*d*) Interleaved controls (*p* > 0.05). Scale bars: 1 mV, 2 ms. (Online version in colour.)

We incubated slices for at least 4 h in 100 μM NG-nitro-l-arginine (L-NNA), an inhibitor of NOS, prior to and during LTP experiments. Tetanus-induced LTP was no different in treated slices (*p* < 0.05, *n* = 8; figure 5*a*) than in interleaved controls (*n* = 11; figure 5*b*).

Another candidate retrograde messenger that has been postulated to mediate plasticity at glutamatergic synapses on stratum radiatum interneurons is the eicosanoid 12-hydroperoxyeicosatetraenoic acid (12-(S)-HPETE), acting on presynaptic transient receptor potential vanilloid 1 channel (TRPV1) receptors [33]. Although in stratum radiatum interneurons this cascade has been reported to mediate LTD induction, in several other brain circuits presynaptic TRPV1 receptor activation facilitates glutamate release [34–36]. Numerous endogenous ligands can gate TRPV1 receptors, including anandamide and lipoxygenase derivatives of arachidonic acid [37–45]. These substrates can be released upon group I mGluR activation [33,40,46,47] and group I mGluR activation is a requirement of LTP onto O-LM cells [7,10,11].

To examine whether a similar mechanism may be involved in potentiating excitatory synapses onto putative O-LM cells, we applied the TRPV1 receptor antagonist, 100 nM 5′-iodoresiniferatoxin, prior to and throughout the induction of LTP. Blocking TRPV1 had no effect on LTP (*n* = 10; figure 5*c*) compared with interleaved controls (*n* = 15; figure 5*d*).

We thus obtained no evidence to support a role for either NO or TRPV1 receptors in the induction of tetanus-induced LTP.

## 4. Discussion

The present results add to the evidence that NMDA receptor-independent LTP in stratum radiatum interneurons depends on postsynaptic Ca^2+^ and is expressed through a presynaptic increase in glutamate release probability. Although this argues for a retrograde factor, we have found no positive evidence to support a role for two candidate signalling mechanisms that have been invoked previously. Neither blockade of NOS nor of TRPV1 receptors affected LTP.

We focused on ‘horizontal’ interneurons with electrical properties typical of positively identified O-LM cells. We reconstructed only a subset of interneurons but confirmed that their dendritic arborization was confined to stratum oriens, and that they projected an axon to stratum lacunosum-moleculare. Because a positive identification was not available for all interneurons, we refer to the cells in this study as putative O-LM interneurons. Importantly, however, we systematically interleaved all experiments where either BAPTA or NOS or TRPV1 blockade were tested with control experiments. Moreover, we used a control pathway to verify that LTP was confined to the tetanized pathway. Another refinement in this study was to record from interneurons in whole-cell mode, with a pipette solution devoid of polyamines. Although prolonged whole-cell recording profoundly diminishes the success rate for induction of LTP, omitting polyamines removes the preferential requirement for hyperpolarization during presynaptic stimulation (anti-Hebbian LTP) [8]. The induction protocol is thus more akin to that used in several other studies that have reported on LTP in stratum oriens interneurons [7,10]. For the purpose of this study, we suggest that the relatively unphysiological induction protocol is justified by the ability to manipulate postsynaptic Ca^2+^ chelation and introduce biocytin without re-patching neurons.

Surprisingly, trains of postsynaptic action potentials could induce a persistent potentiation of transmission. Although cells fired during the LTP induction protocol, the total number of action potentials induced by postsynaptic current injection was approximately 10-fold higher. This difference may explain why tetanic stimulation-induced LTP was pathway-specific. Although back-propagating action potentials have been invoked in LTP induction in principal cells, this also requires presynaptic glutamate release to activate NMDA receptors [48]. Postsynaptic Ca^2+^ influx alone only in principal cells elicits a transient potentiation of transmission [49]. In this study, postsynaptic activity was sufficient to induce a long-lasting potentiation in the absence of presynaptic stimulation. However, we cannot rule out the possibility that spontaneous glutamate release during the action potentials was sufficient for minimal activation of Ca^2+^-permeable AMPA receptors and/or group I mGluRs. Postsynaptic hyperpolarization paired with pharmacological group I mGluR activation has also previously been reported to be sufficient to trigger a persistent potentiation in stratum oriens interneurons, which occludes anti-Hebbian LTP [11]. A direct comparison of these results is difficult because this study was carried out using whole-cell patch-clamp recordings. Nevertheless, we tentatively speculate that, in contrast to principal cells, several sources of Ca^2+^ can converge on an LTP-inducing cascade at glutamatergic synapses on stratum oriens interneurons. The difference between these synapses and those on pyramidal neurons may to some extent be explained by the biochemical compartmentalization offered by dendritic spines in the latter.

The NOS inhibitor L-NNA had no effect on LTP induction. Although we did not perform additional control experiments to detect positive effects of L-NNA, this lends no support to the hypothesis that NO acts as a retrograde messenger in this system. Previous studies of LTP in principal cells have reported divergent results regarding the role of NO. Differences in species and strains, developmental stage, temperature and stimulus protocols have been invoked to explain some of the discrepancies [50–54]. Nevertheless, it is generally established that NO synthesis in pyramidal neurons is triggered by Ca^2+^ influx through NMDA receptors [55,56]. LTP in O-LM cells is NMDA receptor independent [13]. While one study reported that an NOS inhibitor prevented LTP induction in alveus/oriens interneurons [32], this form of plasticity also required NMDA receptors. NMDA receptor-dependent LTP is not prominent in stratum oriens interneurons [8], but has been demonstrated in hippocampal interneurons in strata radiatum and pyramidale [21,24,57]. However, ‘Hebbian’ NMDA receptor-dependent LTP in interneurons is not associated with changes in PPR or receptor occupancy, arguing against presynaptic expression [21,24].

TRPV1 receptors have been reported in hippocampal neurons with mRNA, immunohistochemistry and radioligand binding [58–62], although data from a genetically altered TRPV1 reporter mouse suggest that TRPV1 receptors are not abundant [63]. The effect of presynaptic TRPV1 activation on synaptic transmission is also unclear, with some studies reporting a depression of synaptic release [33] through downregulation of voltage-gated Ca^2+^ channels [64,65], and others reporting a potentiation of presynaptic release [34–36]. Although endogenous TRPV1 ligands can be released upon group I mGluR activation [33,40,46,47], and group I mGluR activation is required for NMDA receptor-independent LTP [7,10,11], we observed no evidence for TRPV1 involvement in LTP in this study: the blocker 5′-iodoresiniferatoxin, applied prior to and during the induction protocol at a concentration previously reported as effective in similar preparations [33], had no effect on LTP.

In conclusion, LTP induction in putative O-LM cells depends on postsynaptic Ca^2+^. It is associated with changes in PPR, failures of transmission and spontaneous EPSP frequency strongly suggestive of presynaptic expression. However, we found no evidence of a role of either NO or TRPV1 receptors in its induction. The retrograde messenger remains to be identified.
